# A Numerical Simulation of Cell Separation by Simplified Asymmetric Pinched Flow Fractionation

**DOI:** 10.1155/2016/2564584

**Published:** 2016-08-15

**Authors:** Jing-Tao Ma, Yuan-Qing Xu, Xiao-Ying Tang

**Affiliations:** ^1^School of Life Science, Beijing Institute of Technology, Beijing 100081, China; ^2^School of Engineering and Information Technology, University of New South Wales, Canberra, ACT 2600, Australia; ^3^Key Laboratory of Convergence Medical Engineering System and Healthcare Technology, The Ministry of Industry and Information Technology, Beijing Institute of Technology, Beijing 100081, China

## Abstract

As a typical microfluidic cell sorting technique, the size-dependent cell sorting has attracted much interest in recent years. In this paper, a size-dependent cell sorting scheme is presented based on a controllable asymmetric pinched flow by employing an immersed boundary-lattice Boltzmann method (IB-LBM). The geometry of channels consists of 2 upstream branches, 1 transitional channel, and 4 downstream branches (D-branches). Simulations are conducted by varying inlet flow ratio, the cell size, and the ratio of flux of outlet 4 to the total flux. It is found that, after being randomly released in one upstream branch, the cells are aligned in a line close to one sidewall of the transitional channel due to the hydrodynamic forces of the asymmetric pinched flow. Cells with different sizes can be fed into different downstream D-branches just by regulating the flux of one D-branch. A principle governing D-branch choice of a cell is obtained, with which a series of numerical cases are performed to sort the cell mixture involving two, three, or four classes of diameters. Results show that, for each case, an adaptive regulating flux can be determined to sort the cell mixture effectively.

## 1. Introduction

Sorting various categories of particles from the mixture to achieve pure sample is of great importance in biological and medical engineering. With the rapid development of micro total analysis systems, small sample volume, high throughput sample processing, high efficiency, and precise particle fractionation are several representative requirements to guide the design of sorting scheme [1]. And correspondingly, a host of particle sorting techniques have been developed in these years: for example, the fluorescence-activated cell sorting [2–4], magnetic-activated cell sorting [5–7], dielectrophoresis sorting [8, 9], and size-dependent sorting [10–12]. The last one has received a remarkable attention attributing to its promising advantages of low cost, high efficiency, and being label-free. There are four typical size-dependent sorting methods that are generally reported, the deterministic lateral displacement [10, 13], the pinched flow fractionation (PFF) [14–16], the cross-flow filtering [17], and the inertial focusing sorting [18]. PFF is relatively simple because there is no extra and specific microstructure needed in the channel, and it has been used to sort polymer beads [14], microparticles [19], and emulsion droplets [20] and for blood cells [21] in recent years. In these above researches, an asymmetric pinched flow fractionation scheme (AsPFF) proposed experimentally first by Takagi et al. [19] is reported to perform a continuous separation and collection for 1.5~5 *μ*m particles; it bettered the traditional PFF remarkably, while there are still some aspects that could be improved, for example, to perform a hydrodynamic analysis and further develop an active and controllable cell or particle sorter.

In the present study, a numerical AsPFF cell sorter model is established with an immersed boundary-lattice Boltzmann method (IB-LBM), where the channel structure, the flow, the multiple sizes of cells, and their interactions are considered. Based on the model, cells with a prescribed size can be manipulated to enter a desired D-branch simply by regulating the flux of one D-branch (or the pressure of one outlet). The numerical results demonstrate that the numerical cell sorter is effective to perform an active and controllable cell sorting, which suggests an improved scheme of AsPFF and is valuable for guiding the experimental design of cell sorter on microfluidic chips.

## 2. Models and Methods

### 2.1. Mathematical Models

In the numerical model, the fluid motion is solved by LBM with D2Q9 lattice model. The discrete lattice Boltzmann equation of a single relaxation time model is [26–28]

(1) gix+eiΔt,t+Δt−gix,t=−1τgix,t−gieqx,t+ΔtGi,

where *g*_*i*_(**x**, *t*) is the distribution function for particles of velocity **e**_*i*_ at position **x** and time *t*, Δ*t* is the time step, *g*_*i*_^eq^(**x**, *t*) is the equilibrium distribution function, *τ* is the nondimensional relaxation time, and *G*_*i*_ is the body force term. In the two-dimensional nine-speed (D2Q9) model [29], **e**_*i*_ are given as follows:

(2) e0=0,0,ei=cos⁡πi−12,sin⁡πi−12hΔt,for  i=1  to  4,ei=cos⁡πi−9/22,sin⁡πi−9/222hΔt,for  i=5  to  8,

where *h* is the lattice spacing. In (1), *g*_*i*_^eq^ and *G*_*i*_ are calculated by [26, 30]

(3) gieq=ωiρ1+ei·ucs2+uu:eiei−cs2I2cs4,Gi=1−12τωiei−ucs2+ei·ucs4ei·f,

where *ω*_*i*_ are the weights defined by *ω*_0_ = 4/9, *ω*_*i*_ = 1/9 for *i* = 1 to 4, and *ω*_*i*_ = 1/36 for *i* = 5 to 8, **u** is the velocity of the fluid, *c*_*s*_ is the speed of sound defined by $c_{s}=h/\sqrt{3}\Delta t$, and  **f** is the body force acting on the fluid. The relaxation time related to the kinematic viscosity of the fluid is in terms of

(4) $$\begin{array}{c} \nu =\left(\;\tau -0.5\;\right)c_{s}^{2}\Delta t. \end{array}$$

Once the particle density distribution is known, the macroscopical quantities, including the fluid density, velocity, and pressure, are then computed from

(5) ρ=∑igi,u=∑ieigi+0.5fΔtρ,p=ρcs2.

Although the lattice Boltzmann method is original from a microscopic description of the fluid behavior, the macroscopic continuity (6) and momentum equations (7) can be recovered from it through the Chapman-Enskog multiscale analysis [31]. Then the LBM maybe can be viewed as a way of solving the macroscopic Navier-Stokes equations:

(6) ∂ρ∂t+∇·ρu=0,

(7) ∂u∂t+u·∇u=−1ρ∇p+ν∇2u+f.

For the IB-LBM frame, the fluid motion is first solved by LBM; then the position of immersed boundary can be updated within one-time step of Δ*t* through [32]

(8) Us,t=∫Ωux,tDx−Xs,tdx,∂X∂t=Us,t,

where **X**(*s*, *t*) is the position of the cell membrane *s* at time *t*. **U**(*s*, *t*) is the membrane velocity and **u**(**x**, *t*) is the fluid velocity. *d* **x** is the lattice side length; *Ω* is the nearby area of the membrane defined by the Delta function *D*(**x** − **X**) [33–35]:

(9) $$\begin{array}{c} D\left(\;x-X\;\right)=\overset{n}{\underset{i=1}{\prod }}\delta \left(\;x_{i}-X_{i}\;\right), \end{array}$$

where

(10) $$\begin{array}{c} \delta \left(\;x_{i}-X_{i}\;\right)=\left\{\begin{array}{cc} \frac{3-2\left|x_{i}-X_{i}\right|+\sqrt{1+4\left|x_{i}-X_{i}\right|-4{\left(x_{i}-X_{i}\right)}^{2}}}{8}, & \left|x_{i}-X_{i}\right|\leq 1,\\ \frac{5-2\left|x_{i}-X_{i}\right|-\sqrt{-7+12\left|x_{i}-X_{i}\right|-4{\left(x_{i}-X_{i}\right)}^{2}}}{8}, & 1<\left|x_{i}-X_{i}\right|\leq 2,\\ 0, & \left|x_{i}-X_{i}\right|>2. \end{array}\right. \end{array}$$

In (9) and (10), *n* denotes the total dimension of the model. The fluid-structure-interaction is enforced by the following equation [27, 32, 33, 36]:

(11) $$\begin{array}{c} f\left(\;x,t\;\right)=\int_{\Gamma }F\left(\;s,t\;\right)D\left(\;x-X\left(\;s,t\;\right)\;\right)ds, \end{array}$$

where **F**(*s*, *t*) is Lagrangian force acting on the ambient fluid by the cell membrane. In the present study, the cell model is proposed as

(12) $$\begin{array}{c} F=F_{l}-F_{b}+F_{s}+F_{e}, \end{array}$$

where **F**_*l*_ is the tensile force, **F**_*b*_ is the bending force, **F**_*s*_ is the normal force on the membrane which controls the cell incompressibility, and **F**_*e*_ is the membrane-wall extrusion acting on the cell. The four force components are [33, 37–39]

(13) Fl=∂∂sKl∂Xs,t∂s−1∂Xs,t∂s,

(14) Fb=Kb∂4Xs,t∂s4,

(15) Fs=KsS−S0S0n,

(16) Fe=KeXs,t−XwXs,t−Xw3,Xs,t−Xw≤rc,0,Xs,t−Xw>rc,

where *K*_*l*_, *K*_*b*_, *K*_*s*_, and *K*_*e*_ are the constant coefficients for the corresponding force components. In (15), *S* is the evolving cell area, *S*_0_ is the reference cell area, and **n** is unit normal vector pointing to fluid. In (16), **X**_*w*_ is the position of the vessel wall, and *r*_*c*_ is the cut-off distance of the effective scope in the membrane-wall interaction.

### 2.2. Physical Model and Simulation Setup

**Figure 1:**
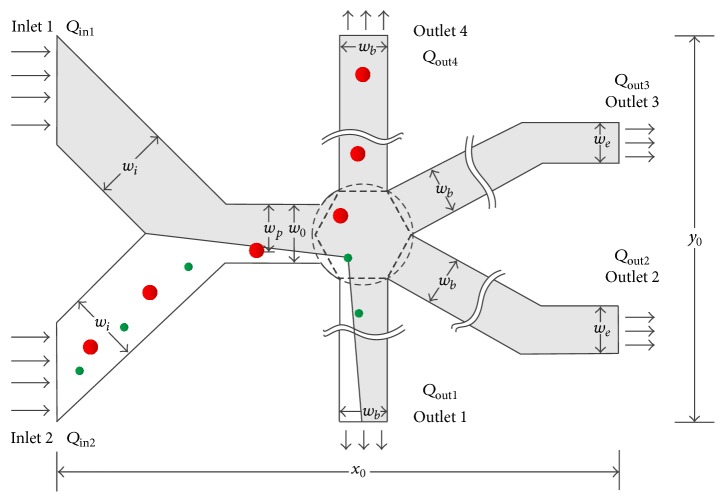
The basic schematic structure of the simulated device.

The geometry model of for cell sorting is illustrated in Figure 1, which consists of 2 upstream branches (U-branches), 1 transitional channel, and 4 downstream branches (D-branches). The U-branches and D-branches branches are labeled with the numbers, as well as the corresponding inlets and outlets. The two U-branches are perpendicular and symmetrical about the center line of the transitional channel. The transitional channel connects the U-branches and a circular buffer area which assembles the entrances of the four D-branches. The D-branches 1 and 4 are straight, while 2 and 3 are folded for the convenience to conduct the boundary condition of outlets; 1 and 4 are also symmetrical about the center line of the transitional channel, as well as 2 and 3. The entire length *x*_0_ and width *y*_0_ of device are 458 *μ*m and 400 *μ*m, respectively. The width of inlet 1 and inlet 2 *w*_*i*_ is 70.71 *μ*m. The width of pinched segment *w*_0_ is 30 *μ*m. The width of outlet 1, outlet 4, and unfolded part of outlets 2 and 3 *w*_*b*_ is 26 *μ*m. The width of folded part of outlets 2 and 3 *w*_*e*_ is 23 *μ*m. *Q* = Δ*p*/*R* is defined as [19], where *Q* is the flux of a D-branch, Δ*p* is the pressure difference between the buffer center and the outlet, and *R* is the flow resistance produced by the microchannel. In order to allocate the flow averagely for all the D-branches under the same pressure boundary conditions, *R*s in all D-branches should be equal. A way to make *R* be equal is described as two steps. First, set the pressure of all outlet to be the same. Second, change the length of the folded part of D-branches 2 and 3 until the stable flows of all outlets are equal. When sorting different size of cells, set the pressure of outlets 1, 2, and 3 to be the same, while the pressure of outlet 4 is regulatable, and the flows of D-branches can be reallocated by altering the outlet pressure. To quantify the the capacity of the reallocation of flow by regulating the flow of outlet 4, we define *β* = *Q*_out4_/(∑_*i*=1_^4^ *Q*_out_*i*__), where bigger *β* means bigger flow through outlet 4 and smaller flow through 1, 2, and 3. In addition, since the flow resistance *R* in each D-branch is the same, the flow *Q* is in proportion to Δ*P*; that is, regulation of flow can be simply realized by regulating the pressure difference; this means that *β* also can be defined as Δ*P*_4_/(∑_*i*=1_^4^Δ*P*_*i*_).

## 3. Results and Discussion

### 3.1. Validation

**Figure 2:**
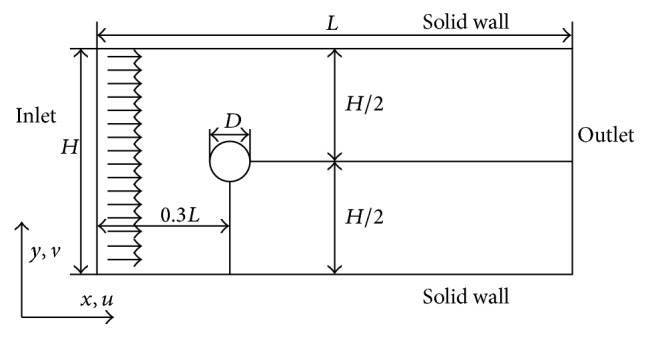
The computational domain for flow past a stationary circular cylinder.

The method and model are validated carefully here by performing a simulation of flow past a stationary circular cylinder. This simulation is carried out by employing IB-LBM model. The computational domain is shown in Figure 2. The length *L* and width *H* of the computational domain are 1000 and 800, respectively. The center point of cylinder is located at *x* = 301 and *y* = 401 and the diameter of cylinder *D* = 40. The cylinder is discretized into a series of points, and the spacing between two adjacent points is 0.6. The cylinder is handled by utilizing immersed boundary method (IB), and the feedback-force principle is adopted to compute the force density on the cylinder, which is described as [22, 40]

(17) Fxs,t=α1∫0tuxs,t−Uxs,tdt+α2uxs,t−Uxs,t,

where **F**(**x**_*s*_, *t*) denotes the interaction force between the fluid and the immersed boundary (cylinder), *α*_1_ and *α*_2_ are large negative free constants, **u**(**x**_*s*_, *t*) is the fluid velocity obtained by interpolation at the IB, and **U**(**x**_*s*_, *t*) is the velocity of the cylinder expressed by **U**(**x**_*s*_, *t*) = *d* **x**_*s*_/*dt*. Here, **U**(**x**_*s*_, *t*) equals 0 because cylinder is stationary. In this case, the ratio of length of the recirculation zone and cylinder diameter *L*_*w*_, the drag force coefficient *C*_*d*_ (18), the lift force coefficient *C*_*l*_ (19), and the Strouhal number *S*_*t*_ are calculated at Reynolds numbers 40 and 100:

(18) Cd=FD0.5ρU∞2D,

(19) Cl=FL0.5ρU∞2D.

The results are shown in Table 1. As shown in Table 1, the present results show close agreements with the general results reported by other literatures. This means the IB-LBM model adopted in present paper is accurate enough.

### 3.2. Determination of the Inlet Flow Ratio *α*

**Figure 3:**
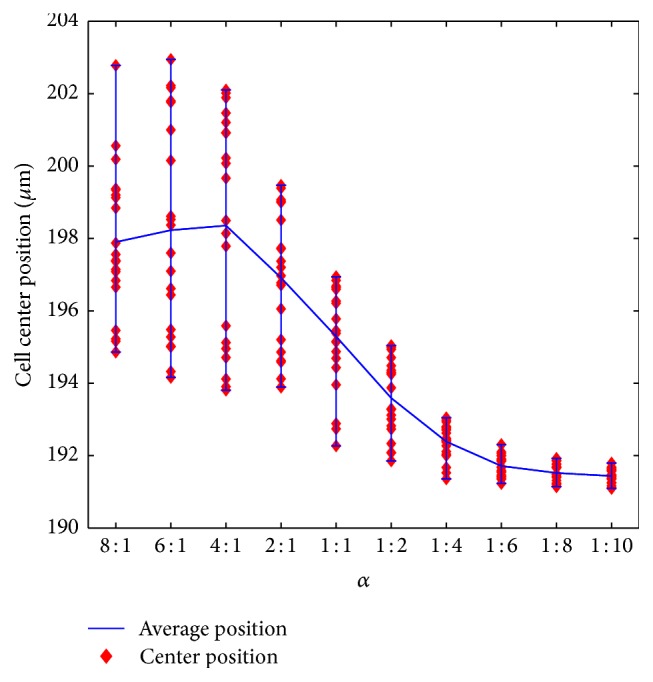
8 *μ*m cell positions in pinched segment at different inlet flow ratio.

In order to actualize the pinched flow to sort cells, it is necessary to establish an appropriate pinched segment in the transitional channel, which is able to lead all cells to move along with the lower sidewall of the transitional channel. There are three aspects for establishing the pinched segment. First, the width *w*_0_ of the transitional channel is better to set as 1.3~1.5 times as the largest diameter of the cells, since it has been proved that a wider *w*_0_ can reduce the fraction effect of pinched flow [14]. Second, the length of the transitional channel is suggested to set as 2 times as *w*_0_; a too long transitional channel may result in central tendency of the flexible cells, which is unfavourable to control the cells to move along with the lower sidewall. Finally, the inlet flow ratio *α* = *Q*_in1_ : *Q*_in2_ is also important to achieve the effective cell sorting. To get a proper *α*, a set of numerical cases are performed by setting *α* = 1/8,1/6,1/4,1/2,1, 2,4, 6,8, and 10, where 20 cells with 8 *μ*m diameter (the smallest size) are initialized and randomly placed in the U-branch 2 to test the function of the pinched flow. The cell center positions at the end of the transitional channel are recorded and shown in Figure 3.

As shown in Figure 3, the cell center positions when leaving the pinched segment drop with the increase of *α*, and finally they reach a relatively steady state when *α* > 6. Although *α* = 8 and *α* = 10 seem to be much better, this means much higher shear stress, which may do damage to the cells. Therefore, *α* = 6 is the choice for the present study.

### 3.3. Effect of *β* and Cell Size on D-Branch Choice

In our consideration, specific, multiple classes of cells with different sizes can be sorted if every class enters a D-branch. In this section, the parameter *β* and the cell size are regulated to manipulate a specific-diameter cell to enter one D-branch, and a series of numerical cases are performed to exhibit the relation of *β*, the cell size, and the choice of D-branch. To set up the numerical model, *β* is regulated from 0.1 to 0.9 with an increment of 0.1. Cells with the same initial diameter are released into U-branch 2. For each case of *β*, four sizes of cell diameter are chosen as 8 *μ*m, 16 *μ*m, 20 *μ*m, and 24 *μ*m to make clear which D-branch a specific diameter of cells prefers to enter. In order to eliminate the possible effect of the initial position of the cell to the D-branch choice, in each case, three randomly placed cells are released into the U-branch, and all the D-branch choices are taken into account.

A D-branch choice for a rigid circular particle can be predicted by the following experimental equations [19]:

(20) w0∗1−βNB−1N−1<D2<w0∗1−βNB−1NN=1,2,3,

(21) D2>w0∗1−βNB−1N−1N=4,

where *w*_0_ is the width of pinched segment as marked in Figure 1, *β* is the outflow ratio at outlet 4, *N*_*B*_ is the total number of outlets, and *D* is the particle diameter. According to the above two equations, the particle will enter the *N*th (*N* = 1,2, 3,4) D-branch if *D* ranges in the scope which can be described with (20) or (21), where (20) is for *N* = 1,2, or 3, and (21) is only for *N* = 4.

**Figure 4:**
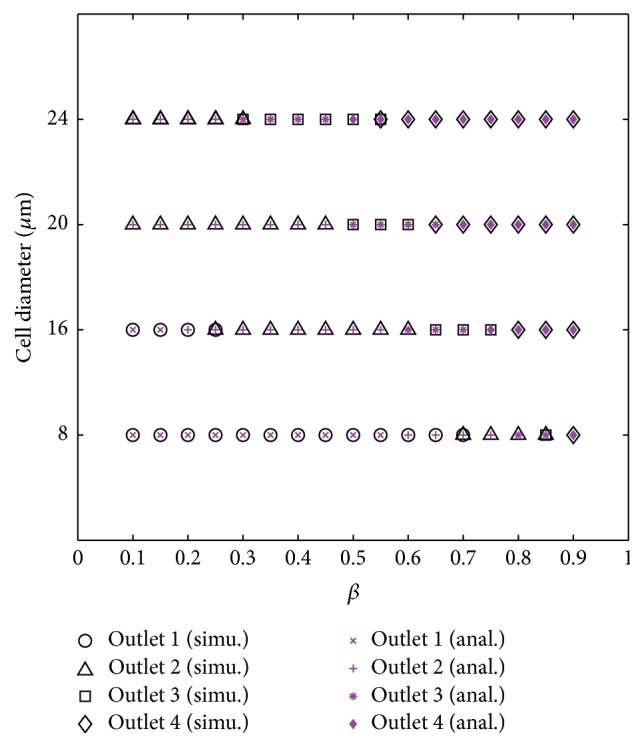
Comparison of simulation and predicted outflow position.

The predicted and numerical results of the choice of D-branch which is related to the cell diameter and *β* are exhibited in Figure 4. In these results, 11 numerical results out of 68 are found not to be consistent to the predicted results, which generally occur at the transition where the cell has approximate probability to enter two neighbouring D-branches. A most possible reason to result in the 11 differences is the predicted results are for rigid particles while cells are flexible.

**Figure 5:**
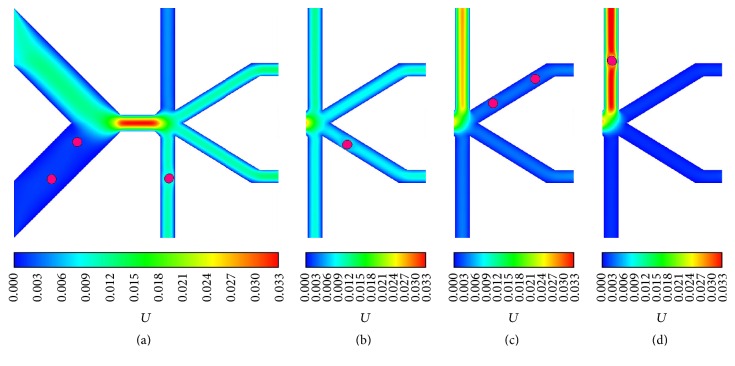
The 16 *μ*m cell outflow positions at different outflow ratios: (a) 0.1, (b) 0.3, (c) 0.7, and (d) 0.9.

**Figure 6:**
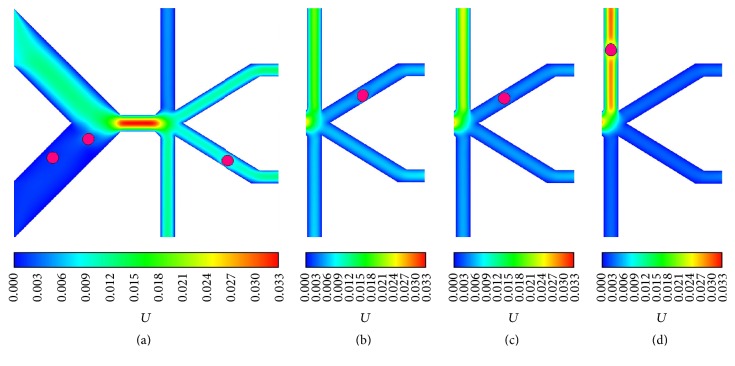
The 20 *μ*m cell outflow positions at different outflow ratios: (a) 0.1, (b) 0.5, (c) 0.6, and (d) 0.8.

According to the results, by regulating *β*, the 8 *μ*m and 16 *μ*m cells can be sent into any one of all four D-branches, and some snapshots of the D-branch choice of 16 *μ*m cell are displayed in Figure 5. By contrast, the 20 *μ*m and 24 *μ*m cells can select one of three D-branches labeled 2, 3, and 4, and the 20 *μ*m cell snapshots are shown in Figure 6. The results indicate that, by simply regulating the flux of one D-branch, cells with the diameters ranging from 8 to 24 *μ*m can be manipulated to enter different D-branches, which gives us an inspiration to sort cells with different sizes if they enter different D-branches at a given *β*.

### 3.4. Size-Dependent Cell Sorting

**Figure 7:**
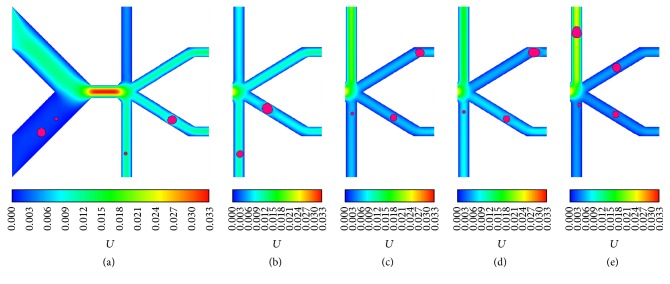
Separation of different-sized cells: (a) separation of 8 *μ*m and 20 *μ*m cells at *β* = 0.1, (b) separation of 16 *μ*m and 24 *μ*m cells at *β* = 0.2, (c) separation of 8 *μ*m, 16 *μ*m, and 20 *μ*m cells at *β* = 0.4, (d) separation of 8 *μ*m, 16 *μ*m, and 24 *μ*m cells at *β* = 0.5, and (e) separation of 8 *μ*m, 16 *μ*m, 20 *μ*m, and 24 *μ*m cells at *β* = 0.6.

As discussed in Section 3.3, cells with different diameters can be manipulated to choose a desired D-branch at a proper *β*; this gives us a potential scheme for sorting cell mixture with different sizes if the cell-cell interaction is not present; that is, all cells in mixture are discrete. In this section, a continuous size-dependent cell sorting is proposed based on the regulation of *β*. According to Figure 4, it is clear which D-branch a certain cell will enter at a specific *β*; therefore, two sizes of cells are sorted once they enter different D-branches. For example, at *β* = 0.1, the 8 *μ*m cell can be sorted from the 20 or 24 *μ*m cell since 8 *μ*m will enter D-branch 1 while the latter two will enter D-branch 2, and the same result will happen if the 8 *μ*m cell is replaced by 16 *μ*m cell. Some corresponding snapshots are shown as in Figures 7(a) and 7(b). By this means, at *β* = 0.4, it can be predicted that three sizes of cell can be sorted, they are 8, 16, and 20 *μ*m cells or 8, 16, and 24 *μ*m cells. Two snapshots of the two cases are displayed as in Figures 7(c) and 7(d), respectively. Especially at *β* = 0.6, the 8, 16, 20, and 24 *μ*m are predicted to enter four different D-branches, and the numerical experiment result validates this actually as exhibited in Figure 7(e).

## 4. Summary and Conclusion

A size-dependent cell sorting model with an asymmetric pinched flow is investigated numerically by immersed boundary-lattice Boltzmann method. In the model, three aspects are summarized as the following. First, the geometry of the channels is designed specially according to the effective cell sorting, where the size of the transitional channel for controlling the pinched segment is discussed in detail. Second, the parameters *α* and *β* are defined, respectively, for the flux ratio of the two inlets and the flux proportion of outlet 4 in all outlets. *α* = 6 is considered as a proper value to prepare for the cell sorting, based on which the regulation of *β* can manipulate cells with different diameters to enter different D-branches. Finally, four sizes of cells are taken into account to exhibit the capacity of cell sorting, and the relations of the regulation flux, the cell size, and the choice of D-branch are analyzed systematically. The simulation results indicate that cells with different diameters can be successfully sorted into different D-branches, this evinces that the model we established is effective, which can provide a directive reference for the design of microfluidic chip for sorting multiple sizes of cells or particles.

## Figures and Tables

**Table 1:** Comparison of the flow characteristics.

Literatures	Re = 40		Re = 100		
	*L*_*w*_	*C*_*d*_	*C*_*d*_	*C*_*l*_	*S*_*t*_
Present	2.40	1.57	1.39	±0.35	0.160
Reference [22]	2.59	1.58	1.39	±0.35	0.160
Reference [23]	2.31	1.57	1.36	±0.34	0.163
Reference [24]	2.35	1.66	1.38	±0.34	0.170
Reference [25]	2.40	1.57	1.40	±0.34	0.162
